# Immune response induced by standard and fractional doses of 17DD yellow fever vaccine

**DOI:** 10.1038/s41541-024-00836-w

**Published:** 2024-03-08

**Authors:** Thais Abdala-Torres, Ana Carolina Campi-Azevedo, Rosiane Aparecida da Silva-Pereira, Luara Isabela dos Santos, Priscilla Miranda Henriques, Ismael Artur Costa-Rocha, Dayane Andriotti Otta, Vanessa Peruhype-Magalhães, Andréa Teixeira-Carvalho, Márcio Sobreira Silva Araújo, Eder Gatti Fernandes, Helena Keico Sato, Francieli Fontana Sutile Tardetti Fantinato, Carla Magda Allan Santos Domingues, Esper Georges Kallás, Helena Tomoko Iwashita Tomiyama, Jandira Aparecida Campos Lemos, Jordana Grazziela Coelho-dos-Reis, Sheila Maria Barbosa de Lima, Waleska Dias Schwarcz, Adriana de Souza Azevedo, Gisela Freitas Trindade, Ana Paula Dinis Ano Bom, Andrea Marques Vieira da Silva, Camilla Bayma Fernandes, Luiz Antônio Bastos Camacho, Maria de Lourdes de Sousa Maia, Collaborative Group for Studies of Yellow Fever Vaccine, Olindo Assis Martins-Filho, Lis Ribeiro do Valle do Antonelli

**Affiliations:** 1 Laboratório de Biologia e Imunologia de Doenças Infecciosas e Parasitárias, Instituto René Rachou, FIOCRUZ-Minas, Belo Horizonte, MG Brazil; 2 Departamento de Bioquímica e Imunologia, Instituto de Ciências Biológicas, Universidade Federal de Minas Gerais, Belo Horizonte, MG Brazil; 3 Grupo Integrado de Pesquisas em Biomarcadores, Instituto René Rachou, FIOCRUZ-Minas, Belo Horizonte, MG Brazil; 4 Faculdade de Ciências Médicas de Minas Gerais, Belo Horizonte, MG Brazil; 5 Divisão de Imunização, Secretaria de Estado de Saúde de São Paulo, São Paulo, SP Brazil; 6 Departamento de Vigilância das Doenças Transmissíveis, Secretaria de Vigilância em Saúde, Ministério da Saúde, Brasília, DF Brazil; 7 External Consultant, Temporary Consulting of the Organização Pan-Americana da Saúde, Brasília, Brazil; 8 Departamento de Doenças Infecciosas e Parasitárias, Escola de Medicina, Universidade de São Paulo, São Paulo, SP Brazil; 9 Secretaria Municipal de Saúde de Belo Horizonte, Belo Horizonte, MG Brazil; 10 Laboratório de Virologia Básica e Aplicada, Instituto de Ciências Biológicas da Universidade Federal de Minas Gerais, Belo Horizonte, MG Brazil; 11 Departamento de Desenvolvimento Experimental e Pré-clínico, Instituto de Tecnologia em Imunobiológicos Bio-Manguinhos, FIOCRUZ, Rio de Janeiro, RJ Brazil; 12 Laboratório de Análise Imunomecular, Instituto de Tecnologia em Imunobiológicos Bio-Manguinhos, FIOCRUZ, Rio de Janeiro, RJ Brazil; 13 Laboratório de Tecnologia Virológica, Instituto de Tecnologia em Imunobiológicos Bio-Manguinhos, FIOCRUZ, Rio de Janeiro, RJ Brazil; 14 Laboratório de Tecnologia Imunológica, Instituto de Tecnologia em Imunobiológicos Bio-Manguinhos, FIOCRUZ, Rio de Janeiro, RJ Brazil; 15 Escola Nacional de Saúde Pública, FIOCRUZ, Rio de Janeiro, RJ Brazil; 16 Departamento de Assuntos Médicos, Estudos Clínicos e Vigilância Pós-Registro, Instituto de Tecnologia em Imunobiológicos Bio-Manguinhos, FIOCRUZ, Rio de Janeiro, RJ Brazil

**Keywords:** Live attenuated vaccines, Cellular immunity, Humoral immunity

## Abstract

The re-emergence of yellow fever (YF) urged new mass vaccination campaigns and, in 2017, the World Health Organization approved the use of the fractional dose (FD) of the YF vaccine due to stock shortage. In an observational cross-sectional investigation, we have assessed viremia, antibodies, soluble mediators and effector and memory T and B-cells induced by primary vaccination of volunteers with FD and standard dose (SD). Similar viremia and levels of antibodies and soluble markers were induced early after immunization. However, a faster decrease in the latter was observed after SD. The FD led to a sustained expansion of helper T-cells and an increased expression of activation markers on T-cells early after vaccination. Although with different kinetics, expansion of plasma cells was induced upon SD and FD immunization. Integrative analysis reveals that FD induces a more complex network involving follicular helper T cells and B-cells than SD. Our findings substantiate that FD can replace SD inducing robust correlates of protective immune response against YF.

## Introduction

Yellow fever (YF) is a vector-borne viral disease caused by an RNA arbovirus from the family Flaviviridae, endemic in the African continent, Central America and South America^1^. In urban areas of Sub-Saharan Africa, the yellow fever virus (YFV) is transmitted by the bite of infected *Aedes aegypti*, and in the Americas, mostly sylvatic mosquitoes (genera Haemagogus and Sabethes) have been involved in the transmission to non-human primates and humans. Large number of cases can occur when the virus is introduced into a populous region with a high density of the vector mosquito. The infection may be asymptomatic, or the symptoms may be nonspecific, including fever, chills, head and body aches, nausea, and vomiting^2^. Disease can evolve to severe forms with icterohemorrhagic and hepatorenal syndromes that are associated with increased mortality^3^.

As a form of prevention, a vaccine composed of attenuated YF virus (17D, 17DD and 17D-213) has been used for over 8 decades^4,5^. It is known that in the first days after vaccination, there is an increase in the number of activated B-cells, consistent with an increase in circulating immunoglobulins and activated CD4^+^ and CD8^+^ T-lymphocytes^6^. In addition, there is an induction of memory CD8^+^ T-lymphocytes^7^ together with a mixed profile of pro and anti-inflammatory cytokines^8^, and long-lasting production of neutralizing antibodies^9^.

Vaccination coverage in endemic regions was reduced, possibly due to a drop in reported cases of the disease, which probably led to the occurrence of major outbreaks in African countries and South America in the last 5 years^10^. Worryingly, these outbreaks were accompanied by many YF cases in previously considered non-endemic regions, which indicated that the virus as well as its vector are being introduced in areas with low or no vaccination coverage^11^.

The attenuated YFV used for vaccine production is expressed in chicken embryos using pathogen-free eggs^12^, whose availability is limited, and thus the doses produced in this process are not enough for mass vaccination campaigns during outbreaks. Therefore, the World Health Organization approved the use of a fractional dose (FD), which consist of one-fifth of the standard dose (SD), as a dose-sparing option for outbreaks response^13^. Indeed, in response to a YF outbreak in the Democratic Republic of Congo in 2016, a mass vaccination campaign employing this fractional dose seroconverted 98% of vaccinees 2 years of age and older^14^. It has been shown that the FD was able to induce neutralizing antibodies against the YF virus^15^ and induced a pro-inflammatory response similar to that generated after the administration of the SD^16,17^. These results indicated that the fractional dose is effective and can be administered. However, little evidence of immunogenicity as compared to viral profile for fractional dose exists for populations in major endemic areas, which would be instrumental to ascertain and recommend the use of fractional doses instead of full doses.

In this article, we have carried out an observational cross-sectional investigation during the large-scale vaccination campaign in the metropolitan area of São Paulo, Brazil. We have compared the viremia kinetics, the production of antibodies, pro and anti-inflammatory mediators and the expansion and activation of T and B-cell subsets after primary vaccination of subjects with the FD or SD of the 17DD-YF vaccine. In addition to providing novel correlates of protection, this study will allow the better understanding of the kinetics of vaccine-induced immune response and the assessment of whether the FD induces humoral and cellular immunological memory similar to the SD.

## Results

### Similar viremia kinetics and comparable seroconversion rates were triggered by primary vaccination with the standard or fractional dose of 17DD-YF vaccine

**Fig. 1:**
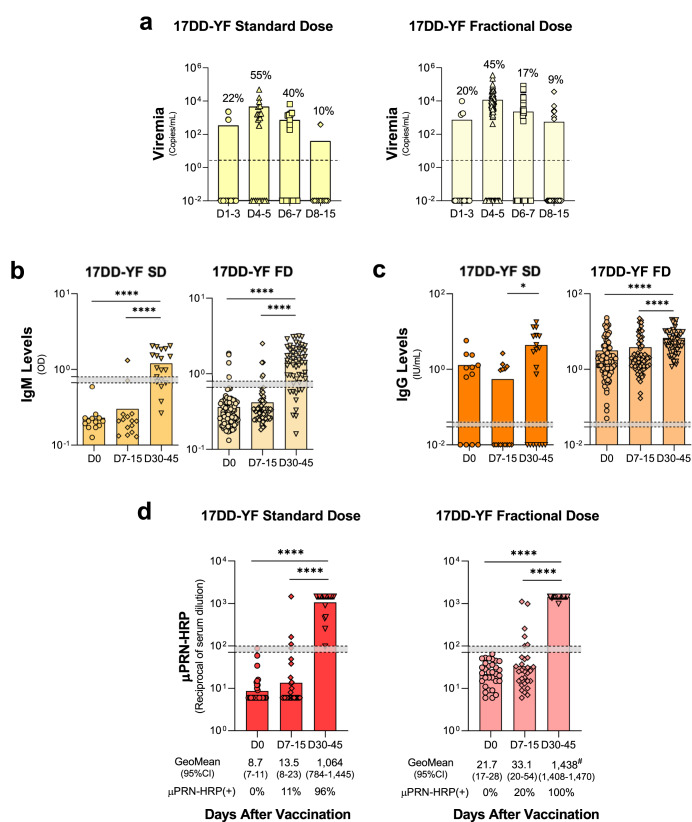
Fractional and Standard doses of 17DD-YF vaccine induce similar viremia kinetics and comparable seroconversion rates. **a** Viremia was quantified by qRT-PCR and data expressed as mean viral copies/mL ( = SD;  = FD). Dashed line represents limit of detection (LOD): 3.45 Log10 copies/mL. **b** YF-specific IgM ( = SD;  = FD) and (**c**) IgG ( = SD;  = FD) were measured in-house ELISA and data expressed as mean OD for IgM and as mean IU/mL for IgG. **d** YF-specific neutralizing antibodies test was quantified by μPRN-HRP assay and data reported as GeoMean of reciprocal of serum dilution ( = SD;  = FD). The results are reported as scattering distribution of individual data at distinct timepoints (D0 / D1-3 = ; D4-5 = ; D6-7 = ; D7-15 / D8/15 = ; D30-45 = ). Dotted lines represent the gray zone. Data above gray background was considered positive. Significant intragroup differences are identified by Kruskal–Wallis test are represented by *p < 0.05; ****p < 0.0001. Intergroups differences in μPRN-HRP GeoMean, identified by Student *t*-test and represented by #.

The viremia and antibody profile were assessed upon SD or FD primary 17DD-YF vaccination (Fig. 1). Data analysis demonstrated that both SD and FD induced similar viremia kinetics with viral copies peaking at D4-5 (Fig. 1a). No significant differences were observed between SD and FD at matching timepoints. However, at D6-7 a smaller proportion of FD vaccinees presented detectable viremia as compared to SD vaccinees (Fig. 1a). The anti-YF IgM titers were similarly induced by SD and FD, with higher levels observed at D30-45 (Fig. 1b). Higher levels of anti-YF IgG were observed at D30-45 in FD vaccinees compared to D0, and in both doses compared to D7-15 (Fig. 1c). The neutralizing antibodies, the gold standard for measuring immunity following vaccination^18^, were measured by micro Plaque-Reduction Neutralization - Horseradish Peroxidase test (μPRN-HRP). The results demonstrated that the primary vaccination with SD and FD induced similar μPRN-HRP seropositivity rates (Fig. 1d).

### A delayed waning in serum soluble mediators was observed in 17DD-YF fractional dose primary vaccinees

**Fig. 2:**
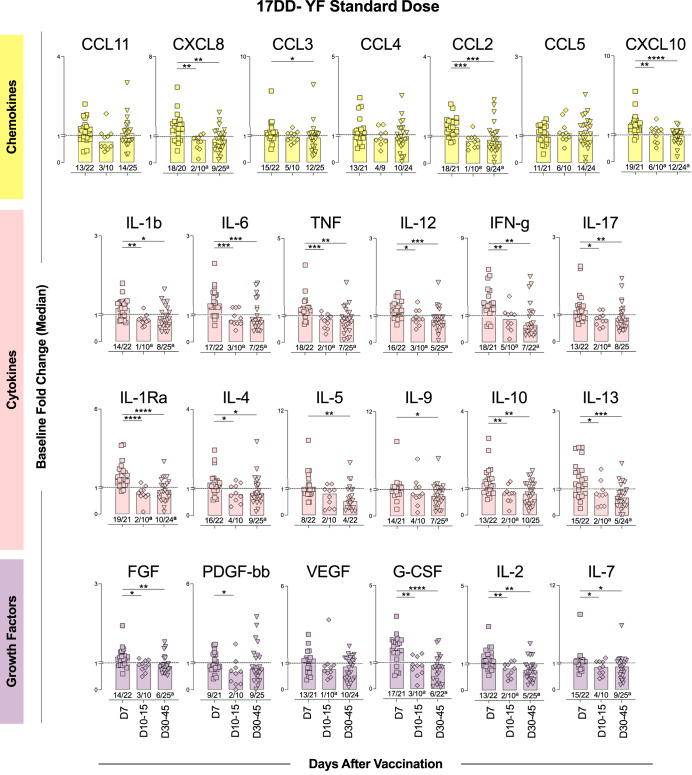
Standard dose of 17DD-YF vaccine elicited an increase of serum soluble mediators with short-term waning upon primary vaccination. Serum soluble mediators (chemokines = ; cytokines =  and growth factors = ) were quantified by high-throughput microbeads array and data expressed as median baseline fold change indices, calculated as described in Materials and Methods. The results are reported as scattering distribution of individual data at distinct timepoints (D7 = ; D10-15 = ; D30-45 = ). Dotted lines represent the cutoff to categorize data as decreased (<1), unaltered (=1) or increased (>1). Only significant differences identified by Kruskal–Wallis test or ANOVA variance analysis were shown. *p < 0.05; **p < 0.01; ***p < 0.001; ****p < 0.0001. Numbers below bars indicate the proportion of individuals with fold changes >1. Chi-square test was used for comparative analysis of proportion of vaccinees with baseline fold change >1 and underscored by the letters “a” and “b” for comparisons with D7 and D10-15, respectively.

**Fig. 3:**
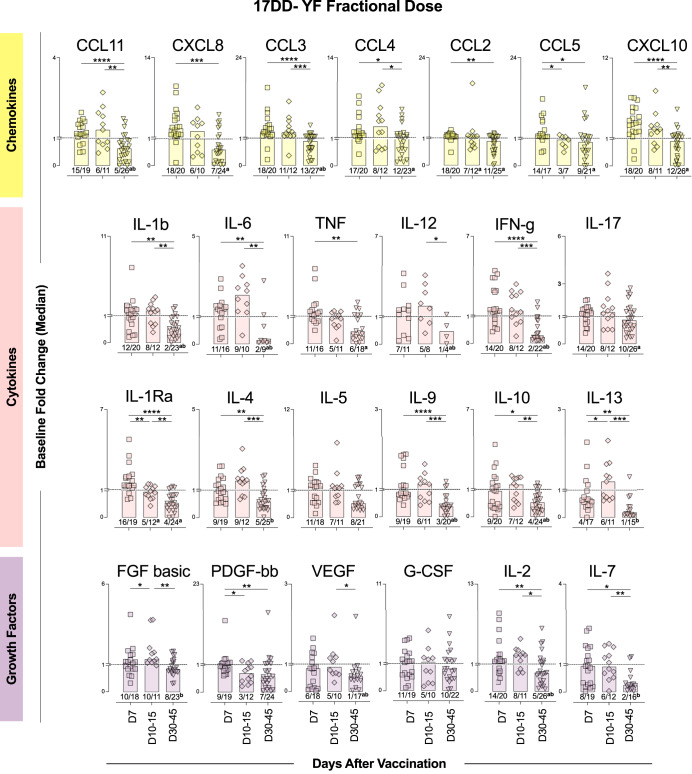
Fractional dose of 17DD-YF vaccine elicited an increase of serum soluble mediators with delayed waning upon primary vaccination. Serum soluble mediators (chemokines = ; cytokines =  and growth factors = ) were quantified by high-throughput microbeads array and data expressed as median baseline fold change indices, calculated as described in Materials and Methods. The results are reported as scattering distribution of individual data at distinct timepoints (D7 = ; D10-15 = ; D30-45 = ). Dotted lines represent the cutoff to categorize data as decreased (<1), unaltered (=1) or increased (>1). Only significant differences identified by Kruskal–Wallis test or ANOVA variance analysis were shown. *p < 0.05; **p < 0.01; ***p < 0.001; ****p < 0.0001. Numbers below bars indicate the proportion of individuals with fold changes >1. Chi-square test was used for comparative analysis of proportion of vaccinees with baseline fold change >1 and underscored by the letters “a” and “b” for comparisons with D7 and D10-15, respectively.

**Fig. 4:**
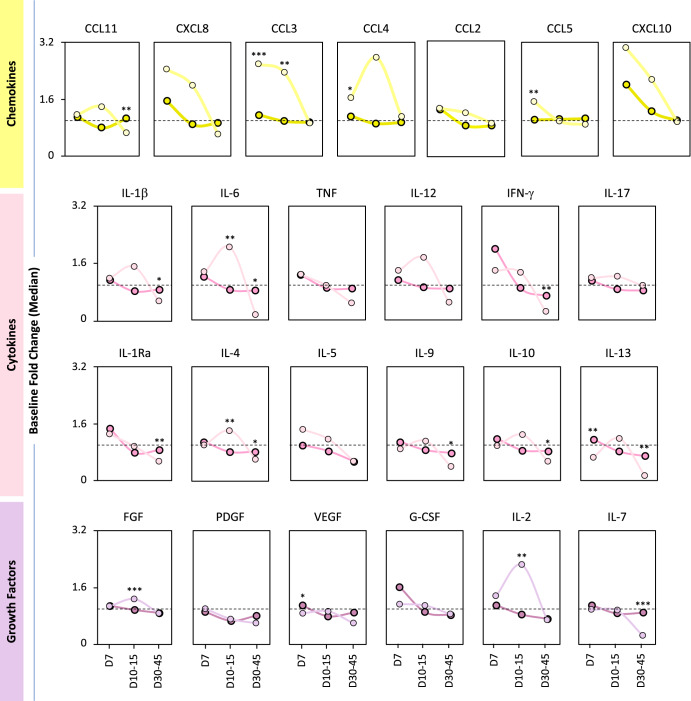
Primary vaccination with Fractional and Standard dose of 17DD-YF vaccine induced a distinct timeline kinetics of serum biomarkers. Serum soluble mediators were quantified by high-throughput microbeads array and data expressed as median baseline fold change indices, calculated as described in Materials and Methods. The results are reported as a line chart expressing the median values observed for FD (chemokines = ; cytokines =  and growth factors = ) and SD (chemokines =  ; cytokines =  and growth factors = ) at each time point. Lines with darker colors represent the Standard dose timeline, and lines with lighter colors denote the Fractional dose timeline. Dotted lines represent the cutoff to categorize data as decreased (<1), unaltered (=1) or increased (>1). Only significant differences identified by Mann–Whitney test were shown. *p < 0.05; **p < 0.01; ***p < 0.001.

Serum soluble mediators (chemokines, cytokines and growth factors) were measured in serum samples from SD and FD vaccinees and the baseline fold changes were determined at distinct timepoints upon primary vaccination (D7, D10-15 and D30-45) (Figs. 2–4 and Supplementary Fig. 2).

Data demonstrated increased baseline fold changes of chemokines (CXCL8, CCL2 and CXCL10), cytokines (IL-1β, IL-6, TNF, IL-12, IFN-γ, IL-17, IL-1Ra, IL-15, IL-10 and IL-13) and growth factors (FGF, PDGF, G-CSF, IL-2 and IL-7) at D7 after SD primary immunization with significant waning observed after D10-15 throughout D30-45. Comparative analysis of the proportion of SD vaccinees with increased production of soluble mediators (baseline fold change > 1) along with the kinetics timeline further confirmed these findings (Fig. 2).

A different profile of soluble mediators was observed in FD primary vaccinees. There was an increase in the baseline fold change for most chemokines (except CCL4) measured at D7, which was maintained elevated at D10-15, with a late waning at D30-45. Elevated levels of cytokines (except IL-12, IL-17 and IL-5) were also observed at D7 and D10-15 compared to D30-45. The analysis of growth factors indicated an increased production of PDGF, IL-2 and IL-7 at D7, with FGF, VEGF, IL-2 and IL-7 presenting higher levels at D10-15 as compared to D30-45. Comparative analysis of the proportion of FD vaccinees with increased production of soluble mediators (baseline fold change > 1) along with the kinetics timeline further confirmed these findings (Fig. 3).

Additional analysis of median baseline fold changes further highlighted the differences between SD and FD vaccinees. The soluble mediators timeline kinetics of SD and FD vaccinees, presented as median baseline fold changes, demonstrated that FD induced a more robust production of soluble mediators, such as CCL3, IL-6, IL-4, FGF and IL-2, compared to the SD, particularly at D10-15 (Fig. 4 and Supplementary Fig. 2A).Venn diagrams were built to identify selective and common attributes along the kinetics timeline upon SD and FD primary vaccination (Supplementary Fig. 2B). At D7, CXCL8 and IL-1Ra presented increased baseline fold change (≥1.5) in both SD and FD. However, IFN-γ and G-CSF were increased selectively in SD as compared to FD. At D10-15, increased baseline fold changes (≥1.5) were observed for IL-6, IL-12, FGF and IL-2 in FD as compared to SD vaccinees. While CXCL10 was commonly induced by SD and FD, CCL3 and CCL4 were observed selectively in FD at D7 and D10 after primary vaccination. Decreased baseline fold changes (<0.5) were observed for IL-6, TNF, IL-12 IFN-γ, IL-9, IL-13 and IL-7 at D30-45 selectively in FD vaccinees.

### The proportion of T-cell subsets expressing activation markers and Tfh cells were higher upon primary vaccination with fractional dose

**Fig. 5:**
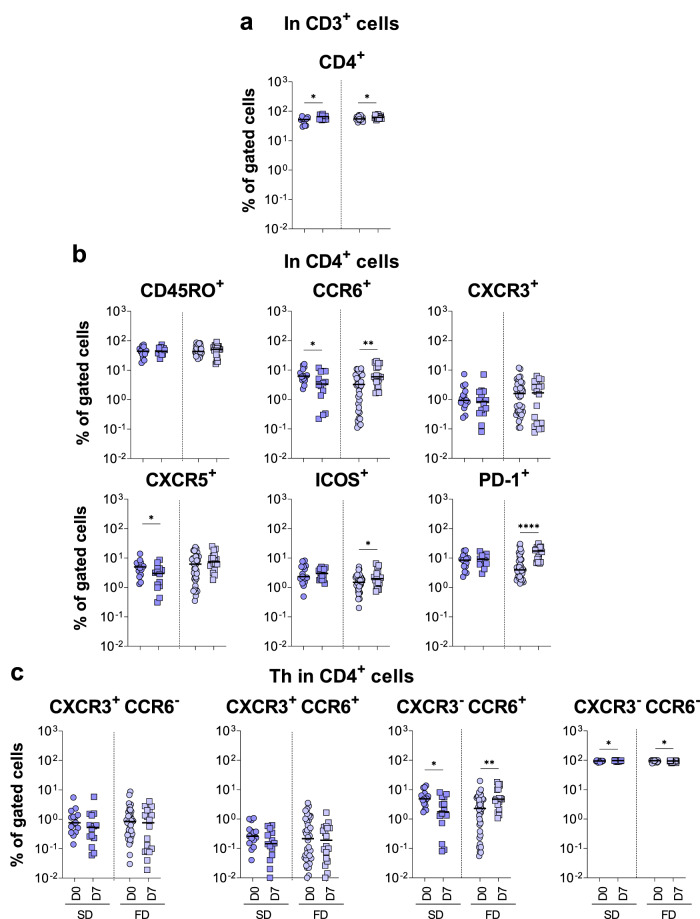
Fractional dose of 17DD-YF vaccine induced higher expression of activation markers on CD4 + T-cells. Immunophenotyping of (**a**) CD4 + T-cells, (**b**) activation markers in CD4 + T-cells and (**c**) Th1 (CXCR3 + CCR6-), Th1/Th17 (CXCR3 + CCR6+), Th17 (CXCR3- CCR6+) and Th2 (CXCR3- CCR6-) subsets in CD4 + T-cells were performed (SD = ; FD = ) by flow cytometry as described in Materials and Methods. The results are reported as scattering distribution of individual data at distinct timepoints (D0 = ; D7 = ). Only significant differences identified by Mann–Whitney test or Student *t*-test were shown. *p < 0.05; **p < 0.01; ****p < 0.0001.

**Fig. 6:**
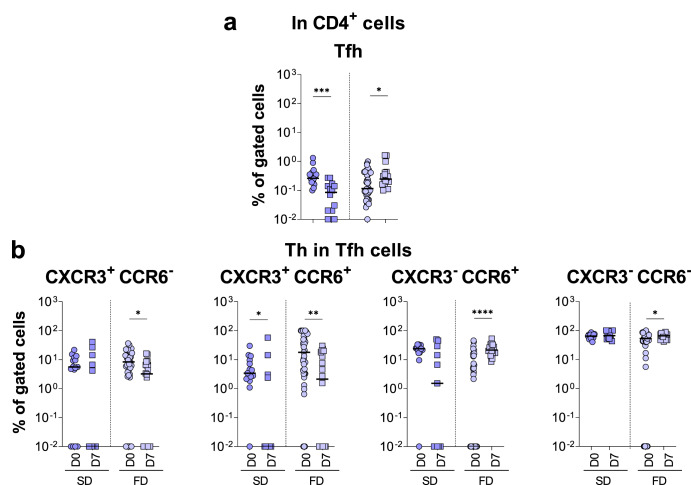
Fractional dose of 17DD-YF vaccine induced higher expansion of T follicular cells. Immunophenotyping of (**a**) Tfh cells and (**b**) Th1 (CXCR3 + CCR6-), Th1/Th17 (CXCR3 + CCR6 + ), Th17 (CXCR3- CCR6+) and Th2 (CXCR3- CCR6-) subsets in Tfh cells were performed (SD = ; FD = ) by flow cytometry as described in Materials and Methods. The results are reported as scattering distribution of individual data at distinct timepoints (D0 = ; D7 = ). Only significant differences identified by Mann–Whitney test or Student *t*-test were shown. *p < 0.05; **p < 0.01; ***p < 0.001; ****p < 0.0001.

**Fig. 7:**
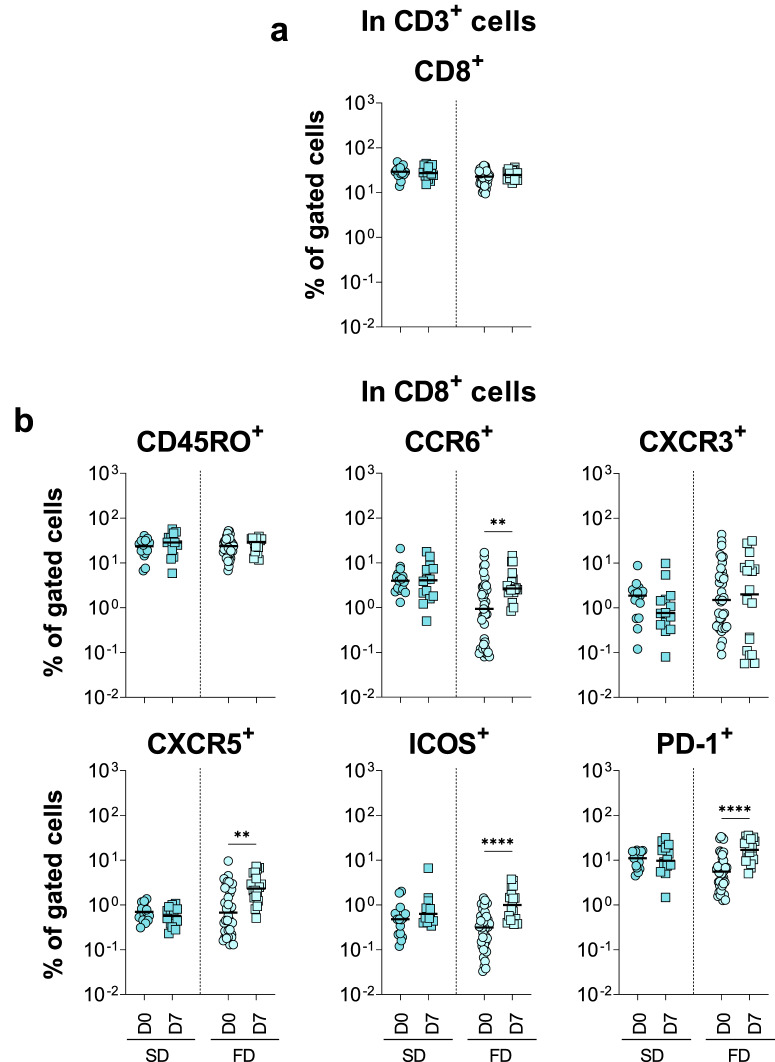
Fractional dose of 17DD-YF vaccine induced higher expression of activation markers on CD8 + T-cells. Immunophenotyping of (**a**) CD8 + T-cells and (**b**) activation markers in CD8 + T-cells were performed (SD = ; FD = ) by flow cytometry as described in Materials and Methods. The results are reported as scattering distribution of individual data at distinct timepoints (D0 = ; D7 = ). Only significant differences identified by Mann–Whitney test or Student *t*-test were shown. **p < 0.01; ****p < 0.0001.

The immunophenotypic analysis of T-cell subsets was performed in peripheral blood samples from SD and FD vaccinees and data compared before (D0) and after (D7) primary vaccination within each dose (Figs. 5–7 and Supplementary Figs. 3 and 4).

Data demonstrated that while both SD and FD doses induced an increase in the frequencies of CD4^+^ T-cells at D7 as compared to D0 (Fig. 5a), decreased frequency of CCR6^+^ and CXCR5^+^ CD4^+^ T-cells were observed in SD vaccinees for the comparisons at the same timepoints. Conversely, increased frequency of CCR6^+^, ICOS^+^ and PD-1^+^ CD4^+^ T-cells were observed in FD vaccinees compared to controls (Fig. 5b). While SD abrogated Th17 and increased the Th2 phenotype amongst CD4^+^ T-cells, an opposite profile was observed in FD vaccinees when comparing D7 to D0 (Fig. 5c).

The analysis of Tfh cells, defined by CD45RO, CXCR5, PD-1 and ICOS expression^19,20^, also demonstrated an opposing pattern, characterized by decreased frequency in SD and increased percentage in FD vaccinees at D7 in comparisons to D0 (Fig. 6a). A progressive increase in Tfh cells was observed at D10-15 in FD vaccinees (Supplementary Fig. 4). Further analysis showed that although both SD and FD vaccinees presented lower frequency of Th1/Th17 cells, FD vaccinees exhibited lower frequencies of Th1 but increased percentages of Th17 and Th2 cells compared to controls (Fig. 6b).

Changes in the CD8^+^ T-cell compartment were observed selectively in FD vaccinees, as demonstrated by increased frequencies of CCR6, CXCR5, ICOS and PD-1 expressing cells (Fig. 7).

### The early changes in the B-cell compartment elicited by the standard dose contrasted with the late profile observed in 17DD-YF fractional dose primary vaccinees

**Fig. 8:**
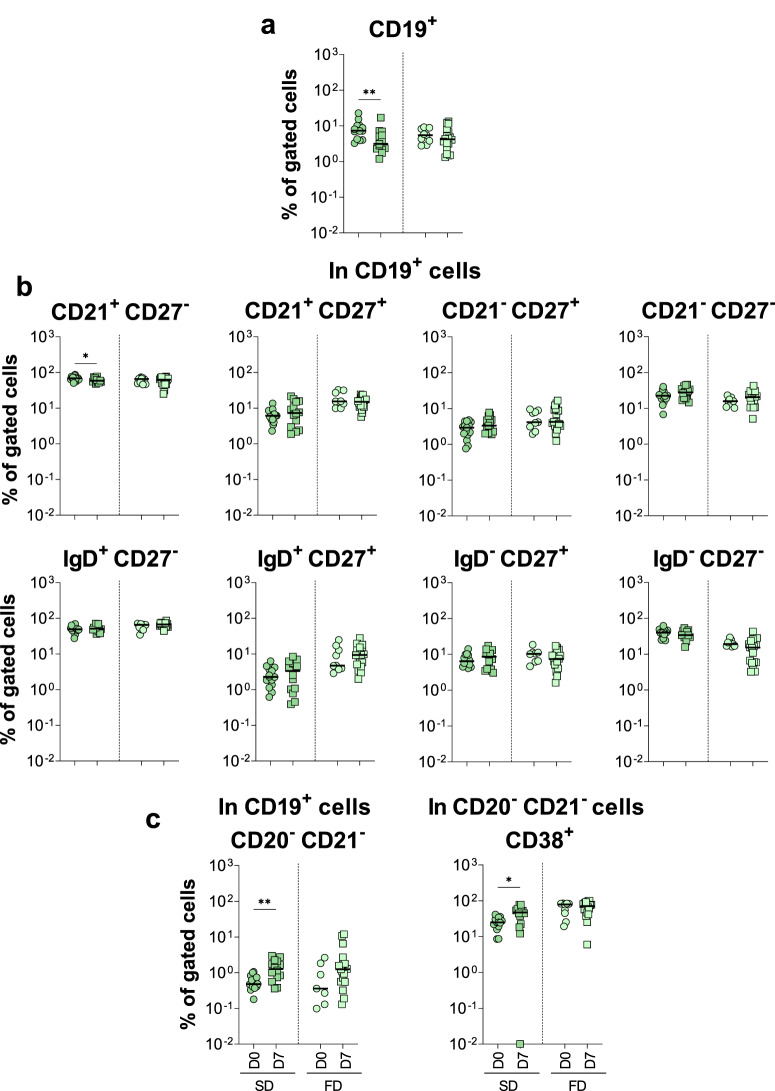
Standard dose of 17DD-YF vaccine induced early changes in the CD19 + B-cell compartment. Immunophenotyping of (**a**) CD19 + B-cells, (**b**) activation markers and isotype-switched memory in B-cells and (**c**) plasma cells were performed (SD = ; FD = ) by flow cytometry as described in Materials and Methods. The results are reported as scattering distribution of individual data at distinct timepoints (D0 = ; D7 = ). Only significant differences identified by Mann–Whitney test or Student *t*-test were shown. *p < 0.05; **p < 0.01.

B-cell phenotypic features were assessed in peripheral blood samples from SD and FD vaccinees before and after primary vaccination (Fig. 8). Few changes were observed in the B cell compartment. Comparative analysis between D0 and D7 demonstrated that SD induced a decrease in the frequency of CD19^+^ cells (Fig. 8a) and naïve (CD21^+^ CD27^−^) B-cells (Fig. 8b). Moreover, higher proportions of plasma cells and activated plasma cells (CD20^−^ CD21^−^ CD38^+^) were observed at D7 in SD vaccinees compared to controls (Fig. 8c). While at D7 no alterations were observed in the B-cell subsets in FD vaccinees (Fig. 8), late at D10-15 a decrease of naïve (IgD^+^ CD27^-^) cells concomitant with an expansion of isotype-switched memory cells (IgD^-^ CD27+) and increased percentage of plasma cells (CD20^-^ CD21^-^) were observed (Supplementary Fig. 4).

### A distinct network connectivity microenvironment involving Tfh cells was induced by primary vaccination with standard and fractional dose of the 17DD-YF vaccine

**Fig. 9:**
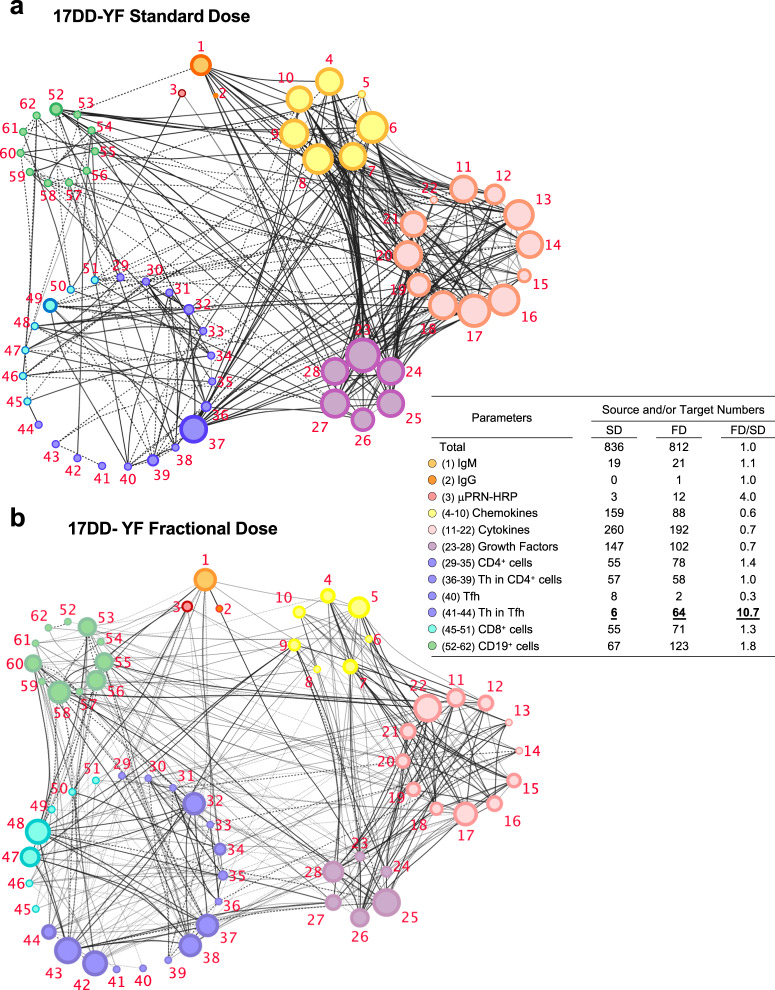
Distinct network connectivity microenvironment was induced by Standard and Fractional doses of 17DD-YF vaccine. Integrative networks were constructed based on correlation scores calculated as described in Materials and Methods. Networks elicited by (**a**) Standard dose and (**b**) Fractional dose were assembled using cluster layouts comprising six groups of parameters including antibodies, chemokines, cytokines, growth factors, T and B-cell phenotypes around D7 after 17DD-YF primary vaccination. Only significant “*r*” scores (*p* < 0.05) identified by Pearson or Spearman rank tests were shown. The node sizes are proportional to the number of correlations between parameters and line thickness illustrates the correlation strength.

The overall snapshot of the integrative network between antibodies, chemokines, cytokines, growth factors and T and B-cell phenotypes were assessed around D7 after SD or FD primary vaccination (Fig. 9, Supplementary Figs. 5 and 6).

Data demonstrated that although both SD and FD induced a similar number of correlations between the parameters (SD, *n* = 836 and FD, *n* = 812), in SD more than half of these correlations involved soluble factors (*n* = 566; chemokines = 159, cytokines = 260 and growth factors = 147) as compared to FD vaccinees (*n* = 382; chemokines = 88, cytokines = 192 and growth factors = 102). Conversely, a higher number of correlations involving cell subsets was observed in FD (*n* = 396; CD4^+^ = 78, Th1/Th17/Th2 in CD4^+^ T-cells = 58, CD8^+^ = 71 and B-cells = 123) as compared to SD vaccinees (*n* = 248; CD4^+^ = 55, Th1/Th17/Th2 in CD4^+^ T-cells = 57, CD8^+^ = 55 and B-cells = 67) (Fig. 9, Supplementary Fig. 5). Noteworthy was that ten times more correlations involving Th in Tfh cells were observed in FD (Th in Tfh = 64) as compared to the SD (Th in Tfh = 6) (Fig. 9a). Conversely, only a few and diffuse number of correlations comprising Tfh cells were observed in SD. While lower connectivity amongst Tfh, T and B-cells was detected for SD (*n* = 8; CD4^+^ = 2, Th1/Th17/Th2 in CD4^+^ T-cells = 2, CD8^+^ = 2 and B-cells = 2), a higher number of correlations (*n* = 37; CD4^+^ = 6, Th1/Th17/Th2 in CD4^+^ T-cells = 6, CD8^+^ = 10 and B-cells = 15) was identified for FD vaccinees (Supplementary Figs. 5 and 6).

## Discussion

The YF vaccine is considered one the most successful vaccines ever developed, being safe, affordable, and able to induce long lasting immunity against the disease within 30 days after vaccination^21^. However, the vaccine is produced in eggs and a limited number of doses are produced by few certified institutions, creating a problem during emergency response to YF outbreaks when there is a global vaccine stockpile shortage. Therefore, in this study, the humoral and cellular responses induced by the standard and fractional doses of 17DD-YF were compared.

Live-attenuated 17DD-YF causes low and transient levels of viremia inducing immunogenicity that can last for decades^15,16,22^. It has been shown that viremia induced by SD reaches its peak after 5 days and clears around 15 days after immunization^23^. Our results showed that the SD and FD induced viremia in a small proportion of vaccinees with a similar kinetics, peaking at day 5 after vaccination, corroborating previous studies^15,16^. It is well known that the induction of humoral response is dependent upon viral replication^24^. Considering the standard dose of 17DD-YF, a single article assessed immunoglobulin levels and reported that ~87% of vaccinees produced IgM and 100% IgG at days 27–40 after vaccine administration^25^. We found that 65% and 74% of individuals who were immunized with the SD and FD, respectively, produced IgM. As for IgG, 60% and 100% of vaccinees presented positive results after SD and FD administration, respectively, at day 30–45. We observed that even before vaccination, some individuals produced IgG, and the proportion of individuals producing anti-virus IgG were much lower than the proportion of those producing neutralizing antibodies, indicating that IgG ELISA is not a reliable readout of protection. This finding might be due to previous and unreported YF vaccination or to cross-reactivity with other flavivirus-specific antibodies^26^.

Neutralizing antibodies have been widely assessed as a reliable correlate of protection induced by YF vaccination^27,28^. The 17DD-YF SD induces 85% of seroconversion in children below the age of 2, ~95% in children between 2 and 12 years, and 98–100% of seroconversion in adults 30 days after its administration^29,30^. Similar seroconversion was induced with the use of the FD in this study. Indeed, our previous data demonstrated that up to a ten-fold lower dose of the 17DD-YF was able to induce similar seroconversion when adults were considered^15,16^.

In addition to neutralizing antibodies, components of cellular immunity are also considered biomarkers of protection^26^. Martins et al. postulated that innate and adaptive cell populations are activated upon 17DD-YF vaccination, and a mixed microenvironment of activation/modulation is key to the development of an effective immune response^6,31^. Further studies demonstrated that a balanced cytokine/chemokine response is necessary for a successful and adverse event-free vaccination^16,32,33^. Campi-Azevedo et al. compared cytokine and chemokine production induced by 5 subdoses of the 17DD-YF vaccine^16^. Similarly, the FD and SD were able to induce the production of the chemokines CXCL8, CCL2 and CXCL10. Moreover, we demonstrated that only the FD was able to induce the production of CCL11, CCL3 and CCL5. The production of these chemokines is triggered by inflammation, and the resulting microenvironment contributes to the recruitment of cells from both innate and adaptive immune responses, amplifying the immune response^34^. Importantly, CXCL10 and CCL11 are secreted by several cell types, such as monocytes, neutrophils, NK, and T-cells, in response to IFN-γ^35,36^. Indeed, some innate immunity-associated genes are upregulated early after YF-17D vaccination leading to the activation of the IFN pathway^37^.

It is widely known that the 17DD-YF vaccine induces a pro-inflammatory profile at D7 after its administration, with augmented production of type 1 cytokines, such as IFN-γ, TNF and IL-12^8,16,38^, and higher levels of these cytokines were associated with seroconversion in adults and children upon vaccination with YF-17D^8,39^. Correspondingly, our results show induction in the production of IFN-γ, TNF and IL-12 early after administration of the SD, along with IL-6, which enhances immune responses after vaccination^40,41^ by amplifying leukocyte recruitment and survival^42,43^, as well as upregulating T follicular helper cells differentiation^44^. The FD of the 17DD-YF vaccine induced an increase in the production of 9 out of 12 cytokines assessed, including TNF, IFN-γ and IL-6, and distinct from the SD, the levels of these cytokines remained augmented up to days 10–15. Interestingly, a late recall of IFN-γ, IL-6, IL-9 and IL-13 levels occurs at 30–45 days after the FD administration compared to the SD.

Recently, the production of growth factors was assessed after SD primary vaccination. In the timeframe between 3 and 28 days after immunization, there was an increase in the production of FGF, VEGF and G-CSF in all vaccinees^45^. Our findings revealed an increase in the production of IL-2 and IL-7, along with G-CSF, early after SD primary vaccination. The FD induced an increase in the levels of FGF, PDGF, IL-2, and IL-7, but not G-CSF, which was highly produced by vaccinees who developed adverse events^45^.

As for the cellular components involved in immunity to YF, several studies have focused on the kinetics of phenotypical changes of innate and adaptive cell populations induced by the 17DD-YF. Although studies in mice are scarce due to model’s limitations, it has demonstrated that protection induced by YF-17D vaccination is dependent on both neutralizing antibodies and T-cells. Partial protection was observed in B-cell deficient mice, which became more susceptible when CD8^+^ T-cells were simultaneously depleted^46^. CD4^+^ T-cells are also key in the development of the protective immune response, since either MHC-II or CD40L deficient mice succumb to challenges with the YF virus after vaccination^46^. In humans, changes in lymphocyte subsets were described after administration of vaccines formulated with different strains of YF virus. Both CD4^+^ and CD8^+^ T-cells are prone to proliferate following 17D-YF vaccination^37^ and an expansion in effector memory T-cells was observed after 17DD-YF immunization^47^.

Although similar antibody production is induced by both SD and FD assessed in this study, differences are found in the composition and activation of T and B-cell compartments. Direct comparisons between SD and FD were not made because samples were collected in different periods and fresh cells were analyzed. Vaccination with FD induced augmented frequencies of the activation markers CCR6, ICOS and PD-1 on both CD4^+^ and CD8^+^ T-cells. Accordingly, previous studies suggest the ability of subdoses of yellow fever vaccine to induce a CD8^+^ T-mediated cellular immunity as those observed for the SD vaccinees^48^. These activation markers were not induced by SD immunization; however, it was already described by others that SD of both YF-17D and 17DD-YF activate T-cells with different molecules used as readouts^6,37^.

Higher frequencies of CXCR5-expressing cells were observed only among CD8^+^ T cells upon FD vaccination. However, even though CXCR5-expressing CD4^+^ T-cells were not increased in FD vaccinees, Tfh cells were exclusively induced by FD immunization. It is known that Tfh cells migrate to germinal centers, where they interact with B-cells, inducing their proliferation, class switching and differentiation, in order to generate high-affinity antibody-secreting and memory B-cells^19,49–51^. Thus, Tfh cells are considered an indicator of a successful vaccination-induced protection^52,53^.

The phenotypic classification of CD4^+^ helper T-cells has been well defined using combinations of surface markers, being the expression of CXCR3 and CCR6 the most employed: CXCR3^+^CCR6^-^ defines the Th1 subset^54–56^, CXCR3^+^CCR6^+^ defines non-conventional Th1 or Th1Th17^57,58^, CXCR3^-^CCR6^+^ defines Th17^57,59,60^ and the absence of both receptors defines Th2 cells^58^. Vaccination with different doses altered distinctly the Th17 and Th2 compartments. Although an expansion of Th2 cells was observed after the SD among CD4^+^ T-cells and a contraction after the FD, no significant alteration was seen in the levels of circulating Th2-associated cytokines. However, an increase in IL-4-producing CD4^+^ T-cells has been previously described in SD vaccinees after antigen-specific stimulation^33,38^. This discrepancy may be due to differences in methodological approaches or time post-vaccination. Data in the literature show decreased IL-17 production^61^, corroborating our finding of lower frequencies of Th17 cells upon SD vaccination. Opposite results were observed in FD vaccinees, wherein we observed an increase in Th17 and Th17-like Tfh cells.

The role of B-cells in generating lifelong protection against pathogens is well established, as they differentiate into long-lasting memory and antibody secreting plasma cells^62^, the latter responsible for the production of neutralizing antibodies^63^. Moreover, the generation of high-affinity antibodies is T-cell-dependent having the Tfh cells a key role in this process^19^. Therefore, induction of B-cell proliferation has been considered, along with Tfh cells and antibody titers, as an indicator of an efficient vaccination^64^. Long-lasting alterations in memory B-cells are not frequently observed after YF vaccination^48^. Nevertheless, a few differences were observed in B-cell compartments in this study. Vaccination with SD induced an expansion in plasma and activated plasma cells early after primary immunization, whereas the FD led to a later expansion in isotype-switched memory B-cells as well as the proliferation of plasma cells, corroborating the increase in Tfh cells^65,66^.

A systems biology tool was employed for an integrative correlation analysis between immunoglobulins, chemokines, cytokines, growth factors and lymphocyte populations. The networks generated with data obtained from volunteers vaccinated with the SD and FD reveal similar number, but distinct patterns of correlations. A comprehensive analysis demonstrated that the number of correlations concerning the T and B-cell compartments was higher in the FD when compared to the SD, in addition to an increase in the number of correlations involving neutralizing antibodies. Importantly, more associations were observed between the Tfh compartment and the other parameters evaluated after FD vaccination. It is noteworthy that most correlations concerning the Tfh subsets involved the B-cell compartment. Indeed, it is well known that the interaction between these two populations is crucial to produce high-affinity antibodies and to induce the proliferation of memory B-cells^19,49–51^. On the other hand, SD induced more correlations concerning soluble mediators and the other parameters assessed.

Overall, there was no difference in the viremia profile and the post-vaccination seroconversion pattern in individuals who received SD or FD. However, analysis of soluble mediators and cellular phenotypic aspects demonstrated that the FD induced a more robust immune response, with higher and persistent levels of soluble mediators, higher frequencies of Tfh and Th17 cells, and earlier activation of CD8^+^ T-cells when compared to the SD. On the other hand, the SD induced an increase in plasma cells early at day 7, which was only observed later after FD immunization. It is also evident that FD and SD trigger a different network connectivity microenvironment between parameters involved in the adaptive immune response. Considering that both FD and SD led to similar seroconversion rates, it is possible that differences observed in the levels of soluble mediators production and T cell activation are due to distinct immune response kinetics. Corroborating this hypothesis, we have found that the increase in plasmablasts observed at D7 in SD vaccinees occur at D10-15 in FD vaccinees.

Taken together, our data demonstrate that the SD and FD induce distinct immune response profiles that do not significantly impact the induction of neutralizing antibodies, reflecting the same outcome of protection against the YF virus, and support the FD use in emergency situations. This study fills a significant gap in the literature providing a better understanding of how FD and SD induce different adaptive immune responses leading to the same levels of protection and, thus, indicating that FD could be a reliable sparing-dose option in the occurrence of new outbreaks, or in case of vaccines’ stockpile shortage. Whether these distinct profiles are beneficial or detrimental to the vaccinees are still at large, and could be clarified with further studies focusing on the long-lasting immunity elicited by FD and SD.

We acknowledge the limitations of a study based on non-probabilistic samples of individuals vaccinated with SD and FD in different time periods and health care units. Although vaccination was not conducted in a research setting, primary care units in Brazil have well developed storage, handling and administration procedures of vaccines. Administration of FD (1/5 of SD) involved adaptation of regular procedures and was preceded by intense training of staff. It should also be considered that participants may have differed in unverified characteristics that might affect their immune responses. However, there are explicit recommendations to exclude or postpone vaccination in individuals with acute infections and selected chronic clinical conditions, such as those that include the use of immunosuppressants. Nevertheless, subtle differences in the results across comparison groups should be interpreted with caution.

## Methods

### Study population

This is an observational cross-sectional investigation comparing two cohorts of vaccinees defined by vaccine dosage (fractional and standard doses) carried out in the metropolitan area of São Paulo (SP, Brazil) by the Collaborative Group for Studies of Yellow Fever Vaccine. The study protocol was submitted and approved by research ethics committees at Instituto René Rachou—Fundação Oswaldo Cruz (CAAE: 82357718.5.0000.5091), Instituto Nacional de Infectologia Evandro Chagas/INI—Fundação Oswaldo Cruz (CAAE: 82357718.5.3001.5262), Secretaria Municipal da Saúde de São Paulo - SMS/SP (CAAE: 82357718.5.3003.0086) and Instituto de Infectologia Emílio Ribas—IIER (CAAE: 82357718.5.3002.0061). The study population comprised a non-probabilistic convenience sampling including whole blood specimens (*n* = 322 samples) collected from healthy subjects of both genders (Males = 142 samples; Females = 180 samples), with age ranging from 11 to 65 years (Mean = 41 ± 14), further categorized into two groups referred as Fractional Dose (FD) and Standard Dose (SD). Written informed consent was given from all participants that have joined this study. Only volunteers with negative results of YF-specific neutralizing antibodies prior to vaccination were enrolled in the present study.

The FD group was enrolled between January and July 2018 at primary care medical centers, during the large-scale vaccination campaign with the fractional dose (1/5 of SD) of the 17DD-YF vaccine in three cities at the metropolitan area of São Paulo (Diadema, Mauá and Santo André). This group included 225 samples (Males = 99; Females = 126), with age ranging from 11 to 65 years (Mean = 43 ± 14).

Between April and August 2019, the SD group was selected during the routine 17DD-YF vaccination at primary care medical centers in São Paulo. This group included 97 samples (Males = 44; Females = 53), aged 17–63 years (Mean = 35 ± 12).

### Biological sample collection and processing

Blood samples (10 mL) were collected at baseline (D0) and at distinct timepoints after primary 17DD-YF vaccination (from D1 throughout D15 and at D30-45) by venipuncture using a gel separation vacuum system without anticoagulant. Aiming to assess the kinetics timeline of distinct parameters, categorical day intervals were defined by grouping D1 to D15 depending on the number of samples available.

Blood specimens were processed to obtain serum samples and peripheral blood mononuclear cells (PBMC) as previously described by Reis et al.^30^. Whole blood samples were submitted to centrifugation at 1000–2000 × g for 10 min at room temperature to obtain serum samples, and aliquots stored at −80 °C at Laboratório de Investigação Médica from Universidade de São Paulo (USP) for further viremia quantification, YF-specific IgM, IgG and neutralizing antibodies detection, as well as analysis of soluble mediators. Clots were processed at Laboratório de Biomarcadores from Instituto René Rachou (IRR, FIOCRUZ-Minas) for PBMC isolation. Clots were removed from the gel separation system, sliced into 1–2 mm fragments, and minced with sterile syringe plungers through 100 μm filters attached to 50 mL conical tubes containing RPMI-1640 medium and resuspended with RPMI supplemented with 10% Fetal Bovine Serum to obtain a final volume of 10 mL. The suspension was slowly applied over 5 mL Ficoll-Paque PLUS cushion (1077 g/mL) and submitted to centrifugation at 410 × g for 40 min at room temperature. PBMC were transferred to 50 mL conical tubes and washed once with 15 mL supplemented RPMI-1640 medium by centrifugation at 300 × g for 7 min at 4 °C. Thereafter, the supernatant was discarded and the cell pellet was treated with ammonium chloride solution (155 mM NH_4_Cl, 10 mM KHCO_3_, 0.1 mM EDTA, pH 7.2) for 10 min at room temperature to lyse the remaining red blood cells. Following centrifugation at 300 × g for 7 min at 4 °C, the PBMC pellet was washed once with RPMI-1640 and resuspended in 1 mL. Aliquots of 10 μL were stained with Trypan Blue (0.4%) to estimate cell viability using the Countess Automated Cell Counter. PBMC suspensions were maintained at 4 °C for further use for immunophenotyping staining.

### Viremia quantification

The quantification of YF viral copies in serum samples collected from D3 throughout D15 after primary immunization with SD or FD of 17DD-YF vaccine was performed through Real-Time Quantitative Reverse Transcription PCR (qRT-PCR) at Laboratório de Tecnologia Virológica in Bio-Manguinhos (LATEV, FIOCRUZ), as previously described by Martins et al.^15^. The limit of detection (LOD) of the test was obtained through the validation process of the one step qPCR assay for yellow fever carried out in Bio-Manguinhos. LOD: 6.25 copies/μL or 3.45 Log10 copies/mL. The results were expressed as viral copies/mL according to the standard curve included in each experimental batch.

### Anti-YF capture IgM detection

The quantification of Anti-YF IgM was performed at the Laboratório de Tecnologia Imunológica from Bio-Manguinhos (LATIM, FIOCRUZ) using a standardized in-house capture enzyme-linked immunosorbent assay (ELISA). Briefly, 96-well plates were coated with 75 μL/well of anti-IgM antibody (10 μg/mL) in carbonate-bicarbonate buffer, pH 9.6, and incubated overnight at 4 °C in a humid chamber. Following incubation, the supernatant was discarded and plates were incubated with 200 μL/well of blocking solution [BDS1, prepared as phosphate-buffered saline (PBS), pH 7.2, supplemented with 0.5% Tween-20 (PBS/T1) supplemented and 5% skimmed milk] for 30 min in a humid chamber at room temperature (RT). After 5 times washing with PBS/T1, 50 μL of pre-diluted serum samples and controls (1:200) in PBS/T1 were added together with 50 µL/well of 17DD-YF live-attenuated virus (2 µg/mL). The viral antigen concentration was previously determined during standardization steps using distinct protein concentrations measured by BCA protein assay kit (Pierce®), according to the manufacturer’s instructions. The plate was incubated overnight at 4 °C in a humid chamber, following washing steps, and 50 μL/well of commercially available anti-Flavivirus antibody (6B6C-1) conjugated with Horseradish Peroxidase was added to each well for 1 h at 37 °C. After washing steps, 75 μL/well of 3,3’,5,5’-tetramethylbenzidine substrate solution (TMB) was added to each well and plates were incubated for 10 min before addition of 50 μL/well of stop-solution (2 M H_2_SO_4_). The endpoint measurements were performed at 450 nm. The optical density (OD) values of each sample were subtracted from the negative control. The results were considered positive at OD > 0.800, borderline at OD ranging from 0.800 to 0.667 and negative at OD < 0.667.

### Anti-YF IgG detection

The quantification of Anti-YF IgG was performed at the Laboratório de Tecnologia Imunológica from Bio-Manguinhos (LATIM, FIOCRUZ) using a standardized in-house enzyme-linked immunosorbent assay (ELISA). Briefly, 96-well plates were coated with 50 μL/well of 17DD-YF live-attenuated virus (2.5 µg/mL) in carbonate-bicarbonate buffer, pH 9.6. The viral antigen concentration was previously determined during standardization steps using distinct protein concentrations measured by BCA protein assay kit (Pierce®), according to the manufacturer’s instructions. The microplates were incubated overnight at 4 °C in a humid chamber and were mechanically washed 5 times with 300 μL/well of phosphate-buffered saline (PBS) pH 7.4, supplemented with 0.05% Tween-20 (PBS/T2). The plates were incubated with 100 μL/well of blocking solution [BDS2, prepared as PBS/T2 supplemented with 0.05% bovine serum albumin (BSA), 3% fetal bovine serum (FBS) and 5% skimmed milk], for 1 h at 37 °C. Following, 50 μL of two-fold serial dilution of serum samples (1:20 to 1:160) in BDS2 were incubated for 1 h at RT. The standard curve was constructed using two-fold serial dilution (1–0.015 UI/mL) of commercially available anti-YF serum (YF–NIBSC). After incubation, plates were washed and reincubated with 100 μL/well of anti-Human IgG conjugated with Horseradish Peroxidase (HRP, BD Biosciences) diluted 1:3.000 in BDS2 for 1 h at RT. Following washing procedures, 100 µL/well of TMB solution was added to each well and the plates were incubated for 15 min before addition of 100 μL/well of stop-solution (2 M H_2_SO_4_). The endpoint measurements were performed at 450 nm. The absorbances of the serum sample dilutions were plotted and the standard curve was used to determine the antibody concentration according to the 4-parameter logistic regression, using the software SoftMax Pro®. The results were expressed in IU/mL relative to the reference anti-YF serum standard curve.

### YF-specific neutralizing antibodies test

The analysis of YF-specific neutralizing antibodies was performed at the Laboratório de Tecnologia Virológica at Bio-Manguinhos (LATEV, FIOCRUZ) using the micro plaque-reduction neutralization—Horseradish Peroxidase test (μPRN-HRP), according to Simões M.^67^. Briefly, in 96-well plates, serial dilutions of serum samples (1:6–1:1458) were pre-incubated with 17D-213/77 vaccine virus (~100 PFU/well) for 2 h at 37 °C, 5% CO_2_, and transferred to pre-formed Vero cells monolayer. Carboxymethylcellulose semisolid medium was overlaid on each well and plates were incubated for 48 h at 37 °C, 5% CO_2_. After incubation, cell monolayers were washed and fixed with ethanol/methanol (1:1) solution and incubated with HRP-conjugated monoclonal antibody (clone 4G2) for 2 h at 35 °C, 5% CO_2_, followed by the addition of True Blue Dye substrate. Thereafter, monolayers were washed, dried and photographed using the ScanLab microscope and images were used for the automated counts. The endpoint-neutralizing antibody titers were defined as the last serum dilution that reduced the number of plaques by 50% (μPRN-HRP_50_) as compared to the virus control included in each assay. Seropositivity was defined considering the antibody titer ≥100 as the cut-off.

### Analysis of soluble mediators by microbead array

High-performance microbead array (Bio-Plex Pro^TM^ Human Assay) was used to quantify serum soluble mediators, comprising: chemokines (CCL11, CXCL8, CCL2, CCL3, CCL4, CCL5 and CXCL10), cytokines (IL-1β, IL-6, TNF, IL-12, IFN-γ, IL-17, IL-1Ra, IL- 4, IL-5, IL-9, IL-10 and IL-13) and growth factors (FGF, PDGF, VEGF, G-CSF, IL-2 and IL-7). The assays were performed according to the manufacturer’s instructions. The results were expressed in pg/mL according to the standard curve of each soluble factor.

### Immunophenotyping assay

Immunophenotyping of T and B-cell subpopulations was performed in 96-well plates by incubating 5 × 10^5^ live PBMC with two panels of monoclonal antibodies. The T-cell panel included anti-CD3-Qdot655 (Invitrogen, clone S4.1, dilution: 0.12:100, Cat #: Q10012), anti-CD4-BV605 (BD, clone RPA-T4, dilution: 1:100, Cat #: 562658), anti-CD8-Alexa Fluor 700 (eBioscience, clone RPA-T8, dilution: 5:100, Cat #: 56-0088-42), anti-CD45RO-BV421 (BD, clone UCHL1, dilution: 1:100, Cat #: 562641), anti-CD27-APC-H7 (BD, clone M-T271, dilution: 5:100, Cat #: 560222), anti-CXCR5-Alexa Fluor 488 (BD, clone RF8B2, dilution: 5:100, Cat #: 558112), anti-CXCR3-PE (BD, clone 1C6, dilution: 5:100, Cat #: 557185), anti-CCR6-PerCP-Cy5.5 (BD, clone 11A9, dilution: 5:100, Cat #: 560467), anti-ICOS-PE-Cy7 (Invitrogen, clone ISA-3, dilution: 5:100, Cat #: 25-9948-42) and anti-PD-1-APC (BioLegend, clone EH12.2H7, dilution: 5:100, Cat #: 329908). The B-cell panel comprised anti-CD19-PE-Cy7 (eBioscience, clone: SJ25C1, dilution: 0.20:100, Cat #: 25-0198-42), anti-CD20-BV650 (BD, clone 2H7, dilution: 2:100, Cat #: 563780), anti-CD21-FITC (eBioscience, clone HB5, dilution: 1.25:100, Cat #: 11-0219-42), anti-CD38- PE (BD, clone HIT2, dilution: 2.5:100, Cat #: 555460), anti-CD27-APC-H7 (BD, clone M-T271, dilution: 2.5:100, Cat #: 560222), and anti-IgD-Alexa Fluor 700 (BD, clone IA6-2, dilution: 2.5:100, Cat #: 561302). Cells were incubated for 20 min at room temperature, washed and resuspended in PBS prior to acquisition on a LSR Fortessa Flow Cytometer (BD Biosciences). The FlowJo V10.8.1 (BD Bioscience) software was employed for data analysis using distinct gating strategies. Lymphocytes were selected based on the size and granularity properties within gated single cells. Thereafter, CD4^+^ and CD8^+^ T-cell subsets selected amongst CD3^+^ T cells were further characterized by additional phenotypic features. B-cell were identified as CD19^+^ cells within CD3^-^ followed by further additional phenotypic analysis of cell subsets.

### Statistical analysis

The GraphPad Prism V8.0 (GraphPad-Software) was used for statistical analyses and graphical arts. Data normality distribution was assessed by the Shapiro-Wilk test. Mann–Whitney test or Student *t*-test were used for comparison between two groups. Kruskal–Wallis or ANOVA variance analysis followed by Dunn’s or Tukey post-test were employed for multiple comparisons. In all cases, significance was considered at *p* < 0.05.

The baseline fold change indices were calculated as the ratio between the results observed for individual samples at distinct timepoints after primary 17DD-YF vaccination divided by the baseline values obtained for the same volunteer before vaccination. Chi-square test was used for the comparison of baseline fold changes categorized as decreased (<1), unaltered (=1) or increased (>1). Significance was considered at *p* < 0.05. Venn Diagram analysis, available at (http://bioinformatics.psb.ugent.be/webtools/Venn/), was employed to assess the common and selective serum soluble mediators with increased (>1.5) or decreased (<0.5) baseline fold-change profiles at distinct timepoints.

Correlation analyses were carried out using Pearson and Spearman rank tests. The “*r*” scores of significant correlations (*p* < 0.05) were employed to build correlation matrices and to assemble networks, using the “corrplot” package of the R software (Project for Statistical Computing Version 3.0.1) and the open-source Cytoscape software platform (available at https://cytoscape.org), respectively. Networks were compiled to arrange clusters of antibodies, chemokines, cytokines, growth factors, T and B-cell subsets. Nodes were used to represent each parameter and connecting lines were employed to identify positive (continuous line) and negative (dashed line) correlations. The node sizes are proportional to the number of correlations between parameters. Line thickness illustrates the correlation strength, ranging from weak/moderate (“r” scores from 0.1 to 0.5 or −0.5 to −0.1, thin lines) to strong correlations (“*r*” scores from ≥0.5 or ≤ −0.5, thick lines).

### Reporting summary

Further information on research design is available in the Nature Research Reporting Summary linked to this article.

### Supplementary information

Supplementary Figures

Reporting Summary

## Data Availability

The data that support the findings of this study are available from the corresponding author [L.R.V.A., O.A.M.F. and T.A.-T.] upon reasonable request.
