# Supplementary material for: Immune response induced by standard and fractional doses of 17DD yellow fever vaccine
Source: NPJ Vaccines. 2024 Mar 8;9:54. doi: 10.1038/s41541-024-00836-w (PMC10923915; doi:10.1038/s41541-024-00836-w)
Supplement: Supplementary file 1 — Supplementary Figures [file 41541_2024_836_MOESM1_ESM.pdf]

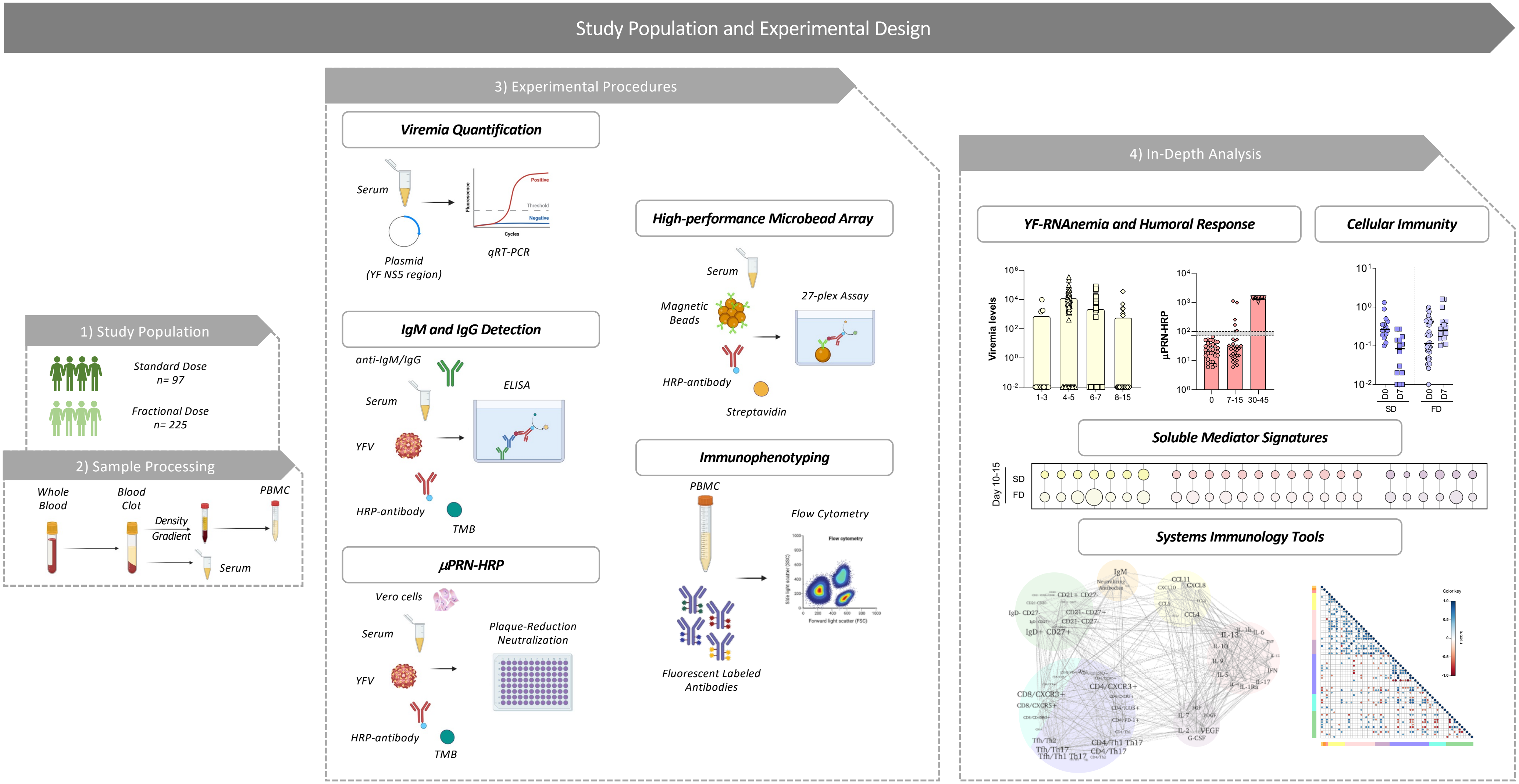


*Supplementary Figure 1– Study population and Experimental Procedures.* The study population was composed by 322 individuals, both sexes, with ages ranging from 11 to 65 years, and was divided in two groups, Standard dose vaccinees (n=97) and Fractional dose vaccinees (n=225). Peripheral blood samples were collected from each volunteer before (D0), from D1 throughout D15 and at D30-45 after primary vaccination, and each sample was processed for obtaining peripheral blood mononuclear cells (PBMC) from blood clots, and serum samples. Serum samples were used in viremia quantification assays by qRT-PCR, YF-specific IgM and IgG titration through ELISA assays, analysis of YF-specific neutralizing antibodies in a micro plaque-reduction neutralization - horseradish peroxidase test (μPRN-HRP) and profiling of serum soluble mediators using a high-performance microbead array. PBMC were used in T and B-cells immunophenotyping to assess effector and memory subpopulations induced by primary vaccination. Results were also employed in a systems immunology tool for analysis of correlation between each data obtained. Flowchart created with BioRender.com.

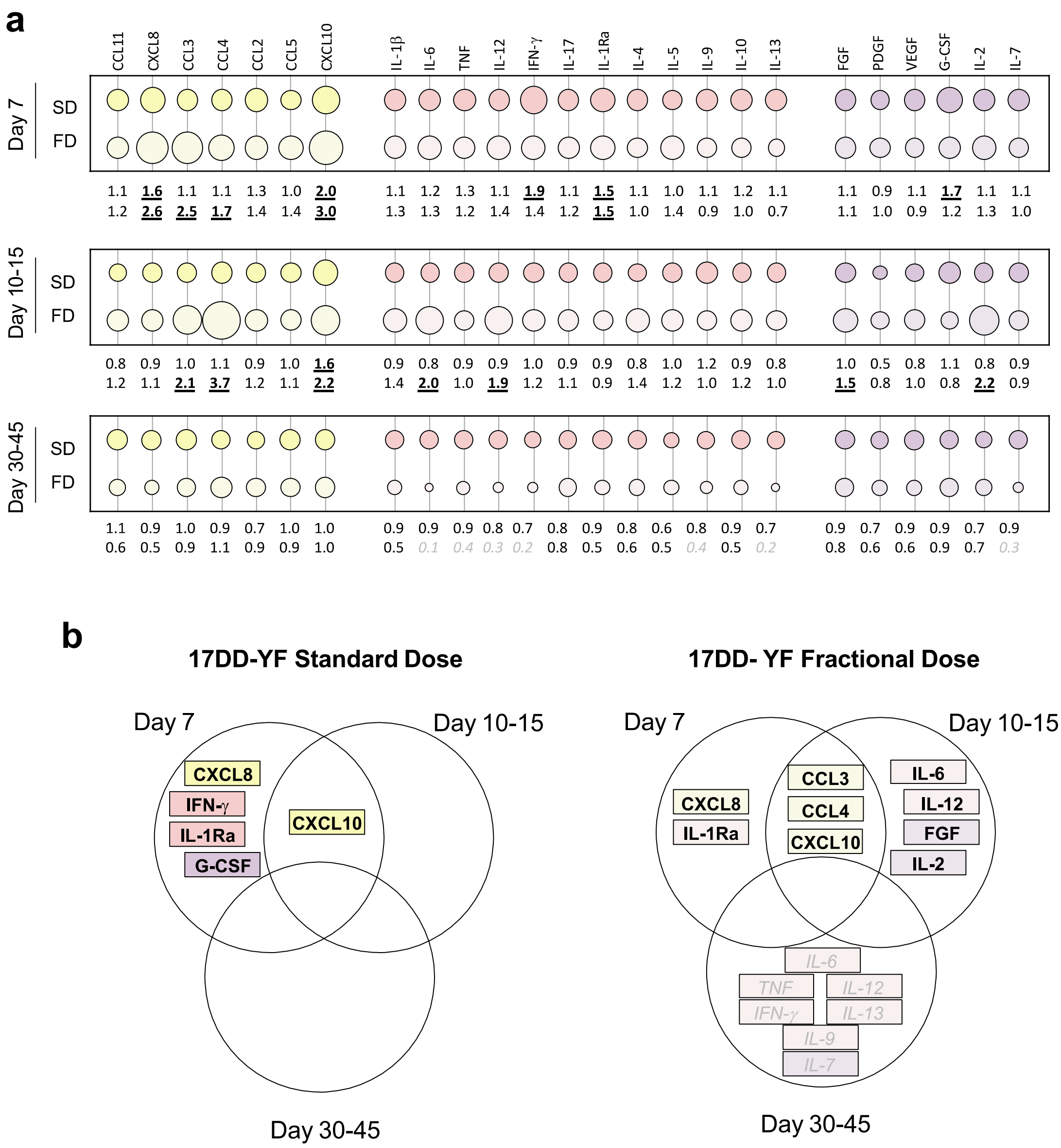


*Supplementary Figure 2 – Fractional and Standard doses of 17DD-YF vaccine elicited increase of serum soluble mediators with delayed waning upon primary vaccination with Fractional dose.* Serum soluble mediators were quantified by high-throughput microbeads array and data expressed as median baseline fold change indices, calculated as described in Materials and Methods. (A) The results are presented in orbital charts reporting the median baseline fold changes of soluble mediators at distinct timepoints upon primary vaccination with Standard (SD) and 1/5 Fractional (FD) dose of 17DD-YF vaccine. Circle size is proportional to the median baseline fold change values provided

below each orbital chart. Fold change values ≥1.5 were considered increased and underlined in bold format, whereas values <0.5 were considered decreased and highlighted in gray italic format. (B) Venn Diagram analysis identify the common and selective serum soluble mediators. Fold change values ≥1.5 were considered increased and underlined in bold format, whereas values <0.5 were considered decreased and highlighted in gray italic format.

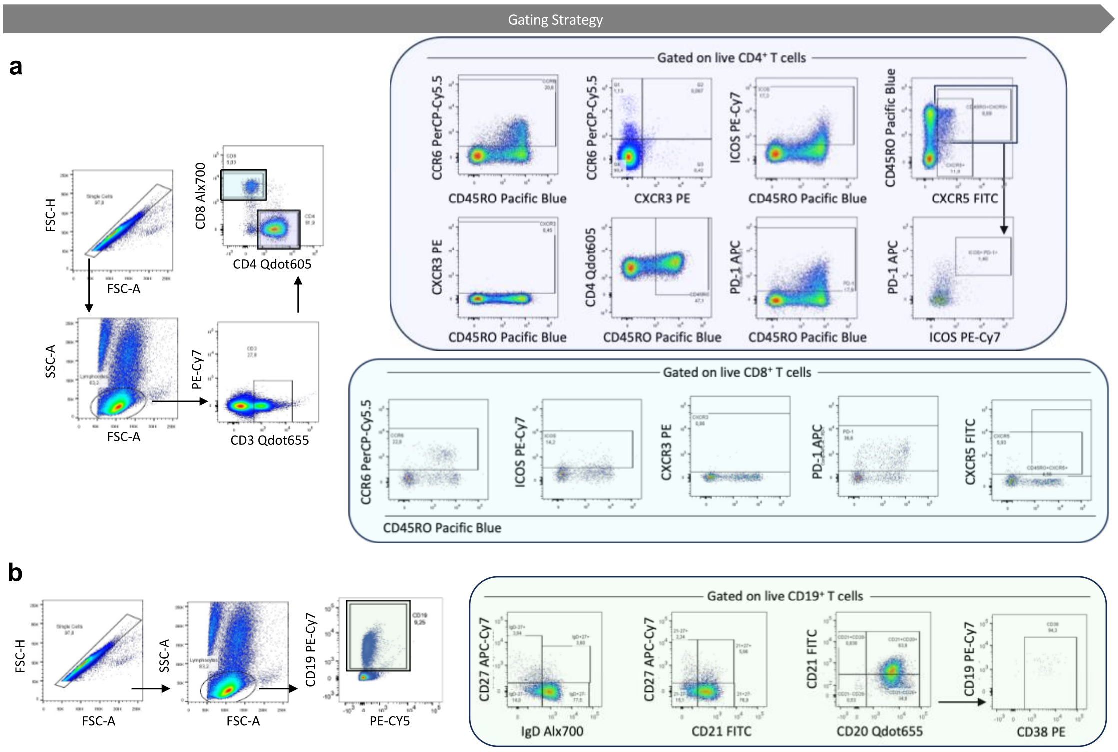


*Supplementary Figure 3 – Manual gating strategy for activation and memory markers on (A) CD4⁺ and CD8⁺ T cells and (B) CD19⁺ B cells.*

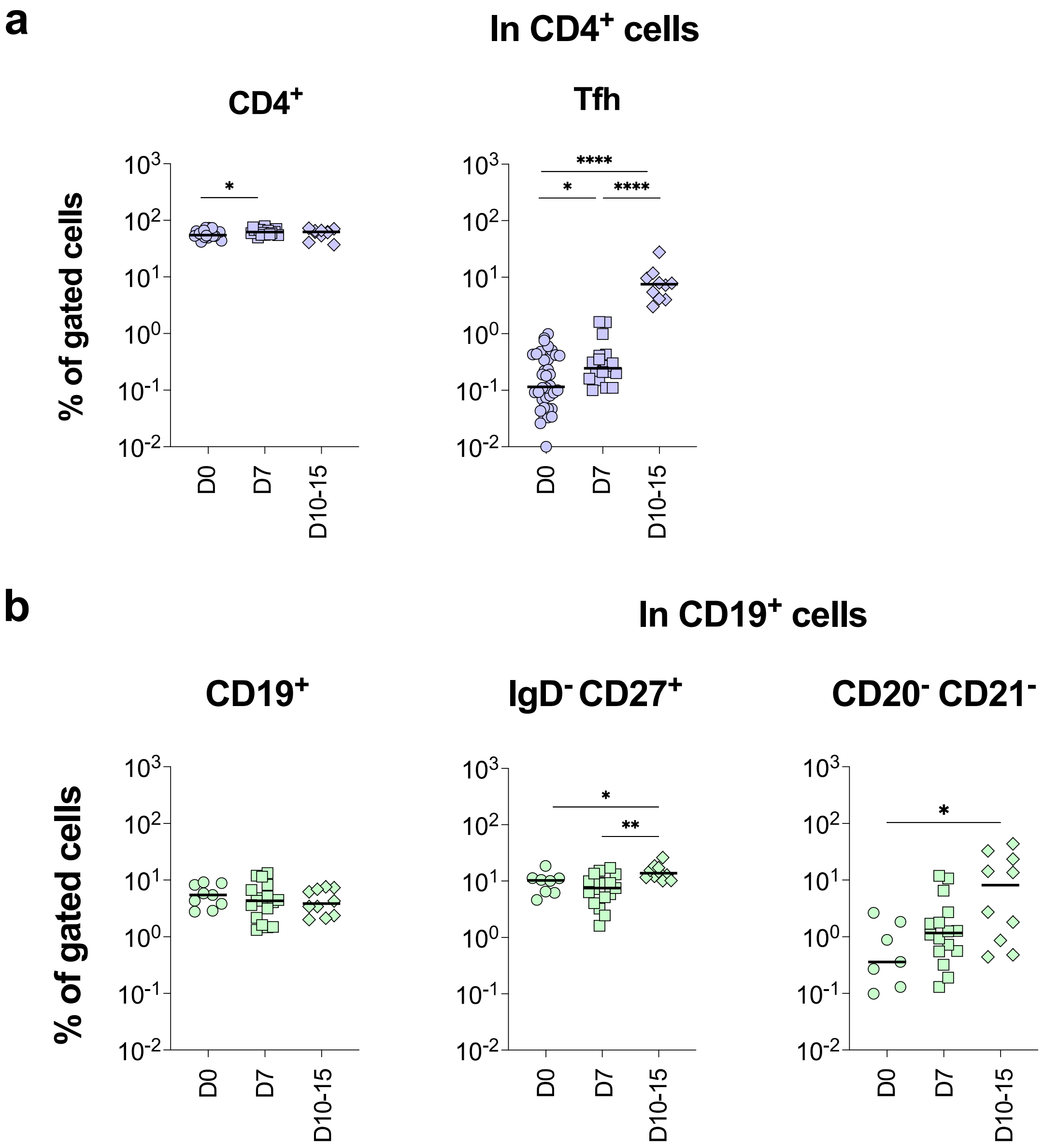


*Supplementary Figure 4 – Primary vaccination with Fractional dose of 17DD-YF vaccine induced a late proliferation of isotype-switched (IgD$^-$ CD27$^+$) B-cells and plasma cells.* Immunophenotyping of (A) CD4$^+$ T-cells and Tfh cells, and (B) CD19$^+$ B-cells and plasma cells were performed by flow cytometry as described in Materials and Methods. The results are reported as scattering distribution of individual data, using symbols to represent distinct timepoints. Only significant differences identified by Mann-Whitney test or Student t-test were shown. *p<0.05; **p<0.01; ****p<0.0001.

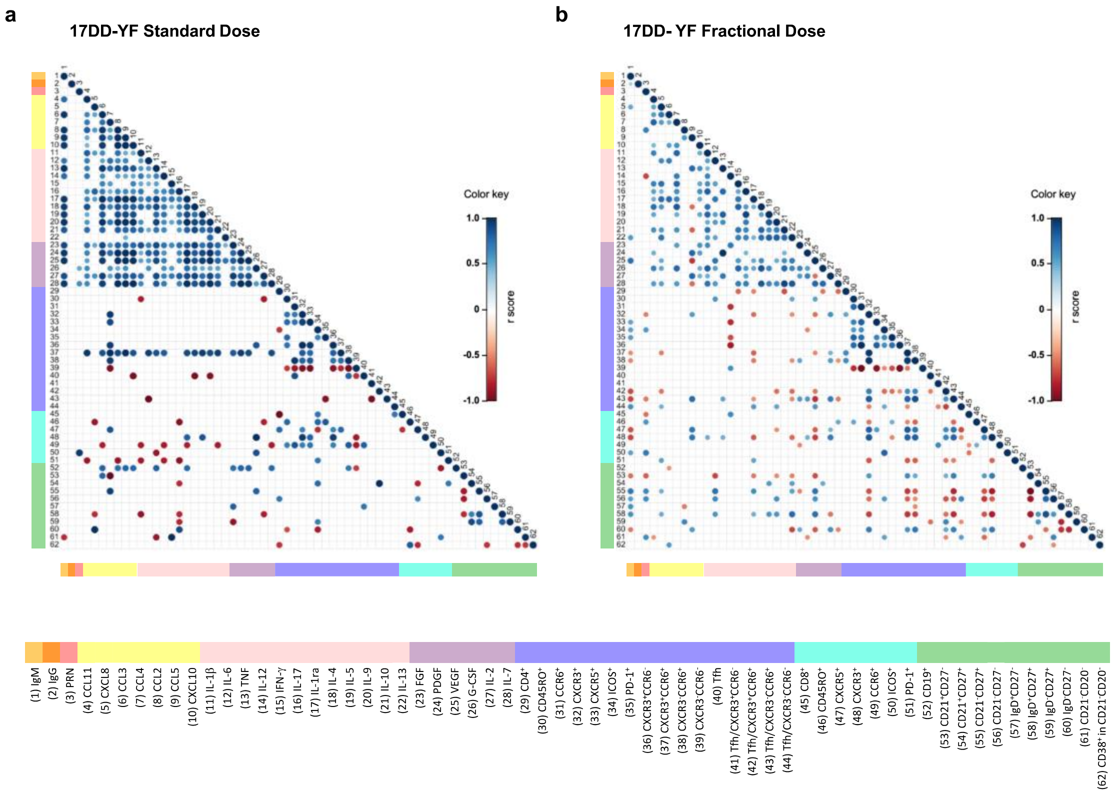


*Supplementary Figure 5 – Fractional and Standard doses of 17DD-YF vaccine induce distinct correlation profile between immunological parameters.* Correlations analysis was employed to build correlation matrices as described in Materials and Methods. Triangle matrices were constructed for (A) Standard dose and (B) Fractional dose comprising sets of parameters including antibodies (IgM, IgG, neutralizing antibodies), chemokines, cytokines, growth factors, $CD4^+$ T-cells, $CD8^+$ T-cells and B-cells around day 7 after 17DD-YF primary vaccination. Only significant "r" scores ($p<0.05$) identified by Pearson or Spearman rank tests were shown, scaled from −1 to +1 according to a gradient color key.

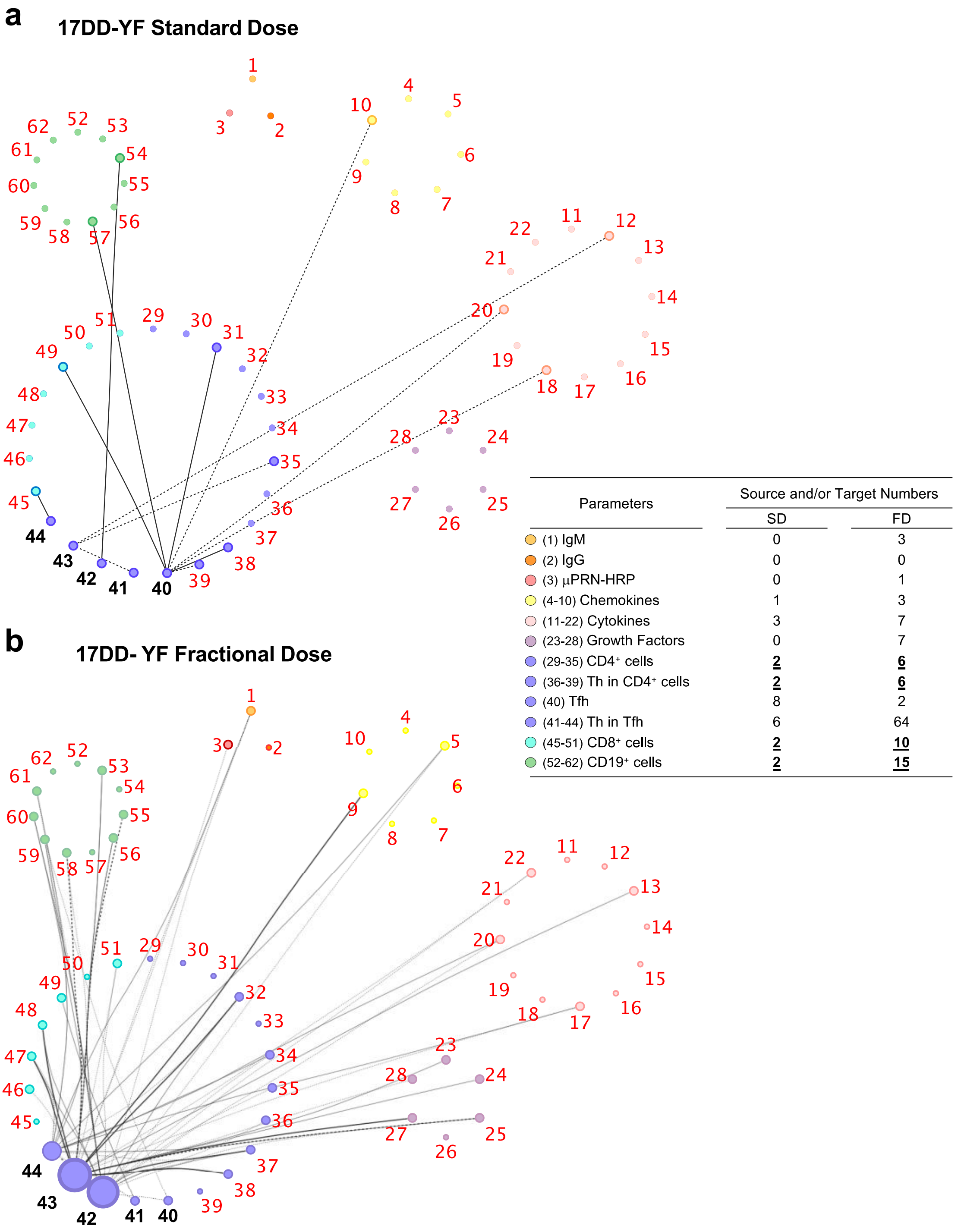


| Parameters | Source and/or Target Numbers | |
|---|---|---|
| | SD | FD |
| (1) IgM | 0 | 3 |
| (2) IgG | 0 | 0 |
| (3) µPRN-HRP | 0 | 1 |
| (4-10) Chemokines | 1 | 3 |
| (11-22) Cytokines | 3 | 7 |
| (23-28) Growth Factors | 0 | 7 |
| (29-35) $CD4^+$ cells | 2 | 6 |
| (36-39) Th in $CD4^+$ cells | 2 | 6 |
| (40) Tfh | 8 | 2 |
| (41-44) Th in Tfh | 6 | 64 |
| (45-51) $CD8^+$ cells | 2 | 10 |
| (52-62) $CD19^+$ cells | 2 | 15 |

*Supplementary Figure 6 – Primary vaccination with Fractional dose of 17DD-YF vaccine induces more correlations comprising Tfh cells and its subsets.* Integrative networks were constructed based on correlation scores calculated as described in Materials and Methods. Networks involving Tfh cells elicited by (A) Standard dose and (B) Fractional dose were assembled using cluster layouts comprising six groups of parameters including:

antibodies, chemokines, cytokines, growth factors, T and B-cell phenotypes around day 7 after 17DD-YF primary vaccination. Only significant “r” scores ($p<0.05$) identified by Pearson or Spearman rank tests were shown. The node sizes are proportional to the number of correlations between parameters and line thickness illustrates the correlation strength.

**Members of the Collaborative Group for Studies of Yellow Fever Vaccine:**

Thais Abdala-Torres[1,2], Ana Carolina Campi-Azevedo[3], Rosiane Aparecida da Silva-Pereira[1], Ismael Artur Costa-Rocha[3], Dayane Andriotti Otta[3], Vanessa Peruhype-Magalhães[3], Andréa Teixeira-Carvalho[3], Márcio Sobreira Silva Araújo[3], Olindo Assis Martins Filho[3], Lis Ribeiro do Valle Antonelli[1,2], Luiz Antônio Bastos Camacho[15], Maria de Lourdes de Sousa Maia[16], Laise Rodrigues Reis[3], Elaine Spezialli[3], Tatiana Guimarães de Noronha[16], Maria Cristina Ferreira Lemos[17], Alexandre Chieppe[18]

[1] Laboratório de Biologia e Imunologia de Doenças Infecciosas e Parasitárias, Instituto René Rachou, FIOCRUZ-Minas, Belo Horizonte, MG, Brazil

[2] Departamento de Bioquímica e Imunologia, Instituto de Ciências Biológicas, Universidade Federal de Minas Gerais, Belo Horizonte, MG, Brazil

[3] Grupo Integrado de Pesquisas em Biomarcadores, Instituto René Rachou, FIOCRUZ-Minas, Belo Horizonte, MG, Brazil

[15] Escola Nacional de Saúde Pública, FIOCRUZ, Rio de Janeiro, RJ, Brazil

[16] Departamento de Assuntos Médicos, Estudos Clínicos e Vigilância Pós-Registro, Instituto de Tecnologia em Imunobiológicos Bio-Manguinhos, FIOCRUZ, Rio de Janeiro, RJ, Brazil

[17] Secretaria de Estado de Saúde, Rio de Janeiro, RJ, Brazil

[18] Superintendência de Vigilância em Saúde – Secretaria Municipal de Saúde do Rio de Janeiro, Rio de Janeiro, RJ, Brazil
