# Supplementary material for: Immune response induced by standard and fractional doses of 17DD yellow fever vaccine
Source: NPJ Vaccines. 2024 Mar 8;9:54. doi: 10.1038/s41541-024-00836-w (PMC10923915; doi:10.1038/s41541-024-00836-w)
Supplement: Supplementary file 2 — Reporting Summary [file 41541_2024_836_MOESM2_ESM.pdf]

nature portfolio

Corresponding author(s): Lis Ribeiro do Valle Antonelli

Last updated by author(s): 01/26/2024

# Reporting Summary

Nature Portfolio wishes to improve the reproducibility of the work that we publish. This form provides structure for consistency and transparency in reporting. For further information on Nature Portfolio policies, see our Editorial Policies and the Editorial Policy Checklist.

## Statistics

For all statistical analyses, confirm that the following items are present in the figure legend, table legend, main text, or Methods section.

| n/a | Confirmed | |
|---|---|---|
| ☐ | ☒ | The exact sample size (*n*) for each experimental group/condition, given as a discrete number and unit of measurement |
| ☐ | ☒ | A statement on whether measurements were taken from distinct samples or whether the same sample was measured repeatedly |
| ☐ | ☒ | The statistical test(s) used AND whether they are one- or two-sided<br>*Only common tests should be described solely by name; describe more complex techniques in the Methods section.* |
| ☒ | ☐ | A description of all covariates tested |
| ☐ | ☒ | A description of any assumptions or corrections, such as tests of normality and adjustment for multiple comparisons |
| ☐ | ☒ | A full description of the statistical parameters including central tendency (e.g. means) or other basic estimates (e.g. regression coefficient) AND variation (e.g. standard deviation) or associated estimates of uncertainty (e.g. confidence intervals) |
| ☒ | ☐ | For null hypothesis testing, the test statistic (e.g. *F*, *t*, *r*) with confidence intervals, effect sizes, degrees of freedom and *P* value noted<br>*Give P values as exact values whenever suitable.* |
| ☒ | ☐ | For Bayesian analysis, information on the choice of priors and Markov chain Monte Carlo settings |
| ☒ | ☐ | For hierarchical and complex designs, identification of the appropriate level for tests and full reporting of outcomes |
| ☒ | ☐ | Estimates of effect sizes (e.g. Cohen's *d*, Pearson's *r*), indicating how they were calculated |

*Our web collection on statistics for biologists contains articles on many of the points above.*

## Software and code

Policy information about availability of computer code

| | |
|---|---|
| Data collection | Flow cytometry data were acquired using LRSFortessa (BD Biosciences). |
| Data analysis | 1. Flow cytometry data were obtained with LRSFortessa flow cytometer and analyzed by FlowJo Software 10.8.1.<br>2. GraphPad Prism version 8 for Mac was used for statistical analysis. The statistical tests used throughout the study are described on methods. |

For manuscripts utilizing custom algorithms or software that are central to the research but not yet described in published literature, software must be made available to editors and reviewers. We strongly encourage code deposition in a community repository (e.g. GitHub). See the Nature Portfolio guidelines for submitting code & software for further information.

## Data

Policy information about availability of data

All manuscripts must include a data availability statement. This statement should provide the following information, where applicable:

- Accession codes, unique identifiers, or web links for publicly available datasets
- A description of any restrictions on data availability
- For clinical datasets or third party data, please ensure that the statement adheres to our policy

The data that support the findings of this study are available from the corresponding author upon reasonable request.

## Research involving human participants, their data, or biological material

Policy information about studies with human participants or human data. See also policy information about sex, gender (identity/presentation), and sexual orientation and race, ethnicity and racism.

| | |
|---|---|
| Reporting on sex and gender | The cohort consisted of 179 females and 143 males.<br>Gender was determined based on self-reporting and no gender-based analyses were performed, because the purpose of this study did not involve disaggregated data. |
| Reporting on race, ethnicity, or other socially relevant groupings | Not applicable |
| Population characteristics | A total of 225 and 97 volunteers vaccinated with fractional and standard dose of the 17DD-YF vaccine respectively were included in the study. The cohorts consisted of 126 and 53 females and 99 and 44 males with a age ranges from 11 to 65 and 17 to 63 years. |
| Recruitment | Subjects were enrolled during vaccination campaigns.<br>Gender: Male & Female<br>Min Age: 11<br>Max Age: 65 |
| Ethics oversight | This study was perfomed under protocols reviewed and approved by the Ethical Committees on Human Experimentation from Instituto René Rachou, Fundação Oswaldo Cruz (CAAE 82357718.5.0000.5091), Instituto Nacional de Infectologia Evandro Chagas/INI – Fundação Oswaldo Cruz (CAAE: 82357718.5.3001.5262), Secretaria Municipal da Saúde de São Paulo - SMS/SP (CAAE: 82357718.5.3003.0086) and Instituto de Infectologia Emílio Ribas – IIER (CAAE: 82357718.5.3002.0061). |

Note that full information on the approval of the study protocol must also be provided in the manuscript.

# Field-specific reporting

Please select the one below that is the best fit for your research. If you are not sure, read the appropriate sections before making your selection.

☒ Life sciences ☐ Behavioural & social sciences ☐ Ecological, evolutionary & environmental sciences

For a reference copy of the document with all sections, see nature.com/documents/nr-reporting-summary-flat.pdf

# Life sciences study design

All studies must disclose on these points even when the disclosure is negative.

| | |
|---|---|
| Sample size | Sample size was non-probabilistic convenience sampling. |
| Data exclusions | Non-primo-vaccinated, based on neutralizing antibody production, were excluded from analysis. |
| Replication | Experiments could not be replicated because we used fresh clot from each volunteer for multiples assays. |
| Randomization | The experimental groups were divided based on before vaccination, time after vaccination and type of vaccine. |
| Blinding | Groups (time point) within each vaccine regimen were blind. Types of vaccine were known because vaccination was carried out in diffent periods. |

# Reporting for specific materials, systems and methods

We require information from authors about some types of materials, experimental systems and methods used in many studies. Here, indicate whether each material, system or method listed is relevant to your study. If you are not sure if a list item applies to your research, read the appropriate section before selecting a response.

## Materials & experimental systems

| n/a | Involved in the study |
|---|---|
| ☐ | ☒ Antibodies |
| ☒ | ☐ Eukaryotic cell lines |
| ☒ | ☐ Palaeontology and archaeology |
| ☒ | ☐ Animals and other organisms |
| ☒ | ☐ Clinical data |
| ☒ | ☐ Dual use research of concern |
| ☒ | ☐ Plants |

## Methods

| n/a | Involved in the study |
|---|---|
| ☒ | ☐ ChIP-seq |
| ☐ | ☒ Flow cytometry |
| ☒ | ☐ MRI-based neuroimaging |

# Antibodies

Antibodies used

anti-CD3 (clone S4.1) - Qdot655, Invitrogen cat# Q10012 Lot: 1863946
anti-CD4 (clone RPA-T4) - BV605, BD cat# 562658 Lot: 7081816
anti-CD8 (clone RPA-T8) -Alexa Fluor 700, eBioscience cat# 56-0088-42 Lot: E10270-1631
anti-CD45RO (clone UCHL1) - BV421, BD cat# 562641 Lot: 6266948
anti-CD27 (clone M-T271) - APC-H7, BD cat# 560222 Lot: 113661
anti-CXCR5 (clone RF8B2) - Alexa Fluor 488, BD, cat#558112 Lot: 7208872
anti-CXCR3 (clone 1C6) - PE, BD cat# 557185 Lot:41714
anti-CCR6 (clone 11A9) - PerCP-Cy5.5, BD cat# 560467 Lot: 9028908
anti-ICOS (clone ISA-3) - PE-Cy7, Invitrogen cat# 25-9948-42 Lot: 4338943
anti-PD-1 (clone EH12.2H7) - APC, BioLegend cat# 329908 Lot: B202982
anti-CD19 (clone: SJ25C1) - PE-Cy7, eBioscience cat# 25-0198-42 Lot: E13454-105
anti-CD20 (clone 2H7) - BV650, BD cat# 563780 Lot: 6258615
anti-CD21 (clone HB5) - FITC, eBioscience cat# 11-0219-42 Lot: E17121-101
anti-CD38 (clone HIT2) - PE, BD cat# 555460 Lot: 2254941
anti-IgD (clone IA6-2) - Alexa Fluor 700, BD cat# 561302 Lot: 4052900

Validation

Titration and fluorescence minus one were used to establish if the reagents were suitable for each use and their concentrations.

# Plants

Seed stocks

*Report on the source of all seed stocks or other plant material used. If applicable, state the seed stock centre and catalogue number. If plant specimens were collected from the field, describe the collection location, date and sampling procedures.*

Novel plant genotypes

*Describe the methods by which all novel plant genotypes were produced. This includes those generated by transgenic approaches, gene editing, chemical/radiation-based mutagenesis and hybridization. For transgenic lines, describe the transformation method, the number of independent lines analyzed and the generation upon which experiments were performed. For gene-edited lines, describe the editor used, the endogenous sequence targeted for editing, the targeting guide RNA sequence (if applicable) and how the editor was applied.*

Authentication

*Describe any authentication procedures for each seed stock used or novel genotype generated. Describe any experiments used to assess the effect of a mutation and, where applicable, how potential secondary effects (e.g. second site T-DNA insertions, mosiacism, off-target gene editing) were examined.*

# Flow Cytometry

## Plots

Confirm that:

☒ The axis labels state the marker and fluorochrome used (e.g. CD4-FITC).

☒ The axis scales are clearly visible. Include numbers along axes only for bottom left plot of group (a 'group' is an analysis of identical markers).

☒ All plots are contour plots with outliers or pseudocolor plots.

☐ A numerical value for number of cells or percentage (with statistics) is provided.

## Methodology

Sample preparation

Peripheral blood mononuclear cells were prepared from clot samples, obtained without anticoagulant, by Ficool-Hypaque density gradient centrifugation (GE Healthcare Life Sciences).

Instrument

LRSFortessa (BD Biosciences).

Software

FlowJo software version 10.8.1 (TreeStar).

| | |
|---|---|
| Cell population abundance | Cell numbers were not limiting in our experiments, since only 2 panels were employed using ex vivo PBMC. |
| Gating strategy | 1. Cells were first gated on a FSC and SSC plot and then gated for singlets (FSC-A vs FSC-H).<br>2. Contamination with neutrophils were excluded using FSC-A vs SSC-A.<br>3. T cells and B cells were gated based on the expression of CD3 and CD19. |

☒ Tick this box to confirm that a figure exemplifying the gating strategy is provided in the Supplementary Information.
